# Molecular diagnosis for screening and elimination of malaria: performance of the first commercially-available malaria LAMP test

**DOI:** 10.1186/1475-2875-11-S1-O30

**Published:** 2012-10-15

**Authors:** Iveth J González, Spencer Polley, Heidi Hopkins, Colin Sutherland, Yasuyoshi Mori, Mark Perkins, David Bell

**Affiliations:** 1 Foundation for Innovative New Diagnostics (FIND), Avenue de Budé 16, Geneva, CH1202, Switzerland; 2 Department of Clinical Parasitology, Hospital for Tropical Diseases, Mortimer Market Centre, Capper St, University London Colleges NHS Foundation Trust, London WC1E 6JB, London, UK; 3 Eiken Chemical Co. Ltd., 1381-3, Shimoishigami, Ohtawara, Tochigi, Japan

## Background

The ability to screen for asymptomatic malaria infection at a field level is increasingly recognized as a key strategy in malaria elimination campaigns. However, molecular methods necessary to detect very low parasite density infections, such as PCR, are restricted to reference-level laboratories and require considerable training to perform. To be effective, such techniques must be close enough to the positive cases to enable rapid treatment. Loop-mediated isothermal DNA amplification (LAMP) is highly sensitive and specific, faster than PCR, requires minimal processing and instrumentation, and allows result detection with the naked eye.

## Materials and methods

FIND has been working with the Hospital for Tropical Diseases in London and Eiken Chemical Company (Japan) in the development of a simplified LAMP assay for the diagnosis of malaria. An optimized test targeting different sequences in the mitochondrial DNA was developed for the detection of parasitaemias below 1 parasite/µl of blood in less than 40 minutes. Prototypes of this test have been compared to PCR with samples from febrile patients in two clinical trials, one in London (travelers) and other in an endemic setting in Uganda.

## Results

Both clinical trials have demonstrated that LAMP is equivalent to nested PCR in sensitivity and specificity with faster time-to-results. In London with 705 samples, sensitivity and specificity of the LAMP *P. falciparum* primers were 98.4% and 98.1% respectively, and for the LAMP Pan primers, 97.0% and 99.2% respectively. In Uganda, 272 samples were tested with the LAMP *P. falciparum* primers and sensitivity and specificity were 93.3% and 85% respectively. This performance of the LAMP assay for malaria was achieved using two simple DNA extraction methods that take only 15 minutes per sample. The study in Uganda also demonstrated that technicians without molecular training could perform the test after a short training period in a simple laboratory space with basic equipment.

## Conclusions

This LAMP test has potential applications both as reference standard for other diagnostics, for primary diagnosis of returned travelers in non-endemic countries, , and as a tool for population screening in malaria elimination campaigns. A high-throughput assay suited to large-scale screening studies is on development.

## Funding

The Foundation for Innovative New Diagnostics (FIND) supports this project through grants from the Bill and Melinda Gates Foundation, the Ministry of Foreign Affairs of the Government of the Netherlands, the UK Department for International Development (DFID), and the Federal Ministry of Education and Research of Germany through the KfW.

